# Comparative analysis of impulsivity profiles in adult Attention Deficit Hyperactivity Disorder and Borderline Personality Disorder

**DOI:** 10.1192/j.eurpsy.2022.2249

**Published:** 2022-09-01

**Authors:** E. Kenézlői, L. Balogh, S. Somogyi, E. Lévay, B. Bajzát, Z. Halmai, P. Soltész, Z. Nemoda, Z. Unoka, R. Tóth, J. Réthelyi

**Affiliations:** 1 Semmelweis University, Department Of Psychiatry And Psychotherapy, Budapest, Hungary; 2 Semmelweis University, Department Of Biochemistry And Molecular Biology, Budapest, Hungary; 3 Hungarian Association for Behavioural, Cognitive and Schema Therapies, Psychology, Budapest, Hungary

**Keywords:** Impulsivity, borderline personality disorder, decision making, Attention Deficit Hyperactivity Disorder

## Abstract

**Introduction:**

High levels of impulsive behavior represent a core symptom of different psychiatric conditions, such as Attention Deficit Hyperactivity Disorder (ADHD), Borderline Personality Disorder (BPD), impulse control and conduct disorders, bulimia nervosa, substance use disorders, and other maladaptive behaviors. Impulsivity is a multidimensional construct, having at least three factors.

**Objectives:**

Our aim was to describe the impulsivity profile in adult ADHD and BPD patients in comparison with a healthy control group, taking into consideration the different impulsivity factors.

**Methods:**

aADHD (n=80) and BPD Patients (n=60) were recruited, based on the DSM-5 criteria. Control subjects (n=80) were screened using Derogatis Symptom Checklist (SCL-90). Comorbidities were assessed by structured clinical interviews. Participants were further investigated with online questionnaires including the Barratt Impulsiveness Scale (BIS-11), Difficulties in Emotion Regulation Scale (DERS), and neuropsychological tests, like Rogers’ decision-making test.

**Results:**

Based ont the BIS-11 and DERS results, significantly higher levels of impulsivity (motor, attentional, non-planning) and difficulties in emotion regulation were present both in the aADHD and BPD groups, compared to the control group. Impulsivity factors were more characteristic to aADHD, emotion dysregulation was more specific to BPD. In the Rogers test, aADHD group was significantly slower in decision-making, while in BPD decision quality and risk-taking were affected.

**Conclusions:**

Impulsivity profiles of the two disorders are different, which leads to the assumption of potentially altered pathway of developing impulsive behavior. As a neuropsychiatric condition, impulsivity in aADHD is related to neurobiological dysinhibition, in BPD impulsive behavior is attached to emotionally involving situations, and emotional dysregulation rooted in childhood adverse events.

**Disclosure:**

No significant relationships.

